# Dietary iron intervention using a staple food product for improvement of iron status in female runners

**DOI:** 10.1186/s12970-014-0050-y

**Published:** 2014-10-18

**Authors:** Ieva Alaunyte, Valentina Stojceska, Andrew Plunkett, Emma Derbyshire

**Affiliations:** Department of Food and Tourism Management Old Hall Lane, Manchester Metropolitan University, Manchester, M14 6HR UK; School of Health Sciences, Liverpool Hope University, Liverpool, L16 9JD UK; College of Engineering, Design and Physical Sciences, Brunel University, Uxbridge, Middlesex UB8 3PH UK

**Keywords:** Iron-deficiency, Athletes, Dietary intake, Ferritin

## Abstract

**Background:**

Adequate nutrient intake is critically important for achieving optimal sports performance. Like all athletes, female runners require a nutritionally balanced diet to maintain daily activities and a successful training regime. This study investigates the effects of cereal product based dietary iron intervention on iron status of recreational female runners (n = 11; 32 ± 7yr; 239 ± 153 minutes exercise/week, of which 161 ± 150 minutes running activity/week; VO_2max_ 38 ± 4 ml/kg/min).

**Methods:**

Participants completed a 6-week dietary intervention study. They were asked to replace their usual bread with iron-rich Teff bread as part of their daily diet. During this period, their dietary habits were assessed by multiple pass 24-hr recalls; iron status was determined by venous blood analysis for serum transferrin, serum transferrin receptor, serum ferritin, total iron-binding capacity and transferrin receptor/ferritin log index.

**Results:**

Pre-intervention a cohort of 11 female runners reported inadequate daily dietary iron intake of 10.7 ± 2.7 mg/day, which was associated with overall compromised iron status. Over a third of all participants showed depleted bodily iron stores (serum ferritin <12 μg/L). Pre-intervention macronutrient assessment revealed adequate energy, protein and fibre intakes, whilst total fat and saturated fat intake was above the recommendations at the expense of carbohydrate intake. A 6-week dietary intervention resulted in significantly higher total iron intakes (18.5 mg/day, *P* < 0.05) and improved iron tissue supply but not enlarged iron stores. Improvements in heamatological indices were associated with compromised baseline iron status, prolonged intervention period and increase in dietary iron intake.

**Conclusion:**

Dietary iron interventions using a staple cereal product offer an alternative way of improving dietary iron intake and favourable affecting overall iron status in physically active females.

## Background

Iron is an essential mineral for optimal physical performance [1]. It has a role in oxygen transport and energy production and serves as a functional component of haemoglobin and myoglobin. Additionally, iron is a crucial part of cytochromes, found in the electron transport system during production of ATP, hence, providing energy source for physical movement. Therefore, in order for the metabolism and oxygen transfer to take place efficiently during physical performance, there must be an adequate level of iron in the human body.

Considerable evidence suggests that nutritional iron deficiency has a negative impact not only on overall health and wellbeing [2] but also on exercise performance [3,4]; hence, improving overall iron status in athletes may enhance their athletic performance. Female athletes, especially runners, are at greater risk of iron deficiency due to increased iron loss in the gastrointestinal tract, sweat, urine and menstruation, and increased haemolysis during endurance training [1]. Furthermore, inadequate dietary iron intake, coupled with limited bioavailability of iron in the diet, may present even a greater risk of iron-deficiency in female athletes.

The effectiveness of conventional iron deficiency treatment of pharmacological doses of iron in supplement form is often questioned due to low compliance rates associated with the side effects such as nausea, stomach pain, constipation and diarrhoea [2]. Therefore, good nutritional practices to achieve adequate iron balance have been suggested as the first line of action in the prevention of iron deficiency in female athletes [3,5]. Although, iron-deficiency anaemia has been shown to have an adverse effect on performance [6,7], the evidence on iron deficiency in the absence of anaemia and the effect on athletic performance is conflicting. Whilst, some researchers reported significant improvements in performance and adaptation in endurance capacity of marginally iron-deficient females after the correction of iron status using iron supplements [8-10], others show no improvement or in fact little adverse effect of marginal iron depletion on exercise performance [11,12].

In addition to this, the research looking at the effects of dietary iron interventions on iron status and exercise performance in female athletes is very scarce. A longitudinal study conducted on swimmers investigated iron status and exercise performance during a 6-month period [13]. The authors reported no differences in iron status or performance scores between iron-rich diet and free choice diet participants. However, a four weeks iron-rich diet, which provided 18.2 mg/d, showed significant effects on serum ferritin concentration in rhythmic gymnasts [14]. Another study reported that a diet rich in iron (11.8 mg iron/day) was more effective in protecting iron status than were the supplements (50 mg ferrous sulphate/day) during 12 weeks of aerobic tests in previous sedentary women [15]. Additionally, the dietary intervention group also showed the highest improvement in their exercise tests. Another study on dietary intervention and iron status in athletes showed that a 4-week dietary advice counselling did not improve overall iron status [16]. However, the authors suggested that diet composition, in particularly the presence of enhancers of non-haem iron absorption, was the most predominant influence for iron absorption in this population. The most recent study investigated the effects of heme iron-rich food product consumption in women of reproductive age [17]. The authors concluded that dietary-based iron treatment using iron-rich crisp bread improved iron status similarly to iron supplements and provided fewer side effects.

The present study aimed to explore dietary iron intervention by the means of a staple food product and the effects of that on iron status in female runners.

## Methods

### Design

This study investigated the relationships between iron-rich food product incorporation into the daily diet and changes of iron status in physically active females during a 6-week intervention period. This study was conducted according to the guidelines laid down in the Declaration of Helsinki and all procedures involving human subjects were approved by the Ethics Committee of a UK Higher Education institution. Written consent was collected from all participants.

### Participants

A total of 15 female subjects, recruited from local running clubs and leisure centers, expressed an interest in participating in the study. Participant’s eligibility was tested by a screening questionnaire. The inclusion criteria included: age 18-45 years, engagement in purposeful physical activity, in particular running, for at least the previous 6 months at the level of at least 30 minutes a day 3 or more times a week, no muscoskeletal problems or recent injury, no heart conditions or complaints, no asthma, normal blood pressure, no-smokers, no current chronic diseases, regular menstrual cycles, no recent iron therapy, no blood donation or haemophilia, no current pregnancy or pregnant within the past year, no food allergies or intolerances, no recent history of eating disorders. After screening, 4 subjects dropped out due to the exclusion criteria (recent pregnancy, n = 1; Diabetes, n = 1) and compliance issues (missed their first appointment, n = 2).

### Procedures

After pre-study screening, eligible subjects were assigned to a 6-week dietary intervention. During the intervention, study participants were asked to swap their usual bread with Teff bread per day (5.6 mg iron per 100 g), which was developed by the research team [18], and not to change any other of their dietary habits or exercise regimes.

All subjects were familiarised with the study’s protocol a week prior to the intervention. Participants’ current diet, exercise levels, anthropometric measurements, blood iron parameters and exercise performance were assessed on baseline (week 0), midpoint (week 3) and end (week 6) of the intervention. Subjects were also asked to keep a bread consumption log in order to record their compliance.

### Anthropometric assessment

Subjects’ height and weight were recorded at baseline (week 1), midpoint (week 3) and end (week 6) of the study using Seca 217 stadiometer (Cranlea, Birmingham, UK) and Seca 711 personal weighing machine (Cranlea, Birmingham, UK), respectively.

### Dietary assessment

Dietary intakes and supplements usage were collected using multiple pass 24-hour diet recalls for each participant at baseline (week 1), midpoint (week 3) and end (week 6). The food records were analysed using NetWisp 3.0 (Tinuviel Software, Llanfechell, Anglesey, UK) diet analysis software. Missing food items’ nutritional data were manually entered into the software by either locating food composition from product manufacturers’ websites or the McCance and Widdowson’s Composition of Foods integrated dataset (6th Summary, UK Nutrient Databank, 2002, UK).

Under-reporting was assessed by Goldberg cut-off limits [19]. Total energy was tested for under-reporting by applying the following formula: Basal metabolic rate (BMR) X 1.55. Test values below the cut-off limit were considered to represent underreporting. BMR for each participant was obtained by Henry equation [20].

Nutritional data were compared to the dietary reference values (DRVs) for energy [21], macro- and micronutrients [22]. Because carbohydrate requirements for athletes are higher than those in general population, carbohydrate recommendation intake was based on the American Dietetic Association (ADA), Dietitians of Canada (DC), and American College of Sports Medicine (ACSM) joint position statement recommendations [23].

### Haematological assessment

Iron status was assessed from non-fasted blood samples taken at baseline (week 0) and completion of the intervention (week 6). To limit the possibility of an acute phase response to exercise affecting these results, samples were taken at least 12 hours after the last exercise session and at the same time of the day followed a controlled fluid and food intake [24].

The following blood indices were analysed: serum ferritin (sFer), serum transferrin (sTRF) and serum transferrin receptor (sTsfR). Because these serum iron status indicators are not immediately influenced by food intake [25], the subjects did not fast before having their blood taken. Serum was prepared from venous bloods by centrifugation after clotting and was stored at -20°C for determination of iron status indices. Serum ferritin (sFer) was measured by immunometry, using an electrochemiluminescence immunoassay (ECLIA) on Modular Analytics E170 (Roche Diagnostics GmbH, Mannheim, Germany). Serum transferrin was measured by immunoturbidimetric assay; and serum transferrin receptor (sTsfR) by particle-enhanced immunoturbidimetric assay, both using Cobas integra system (Roche Diagnostics GmbH, Mannheim, Germany). All serum samples were analysed concurrently at the completion of the study to eliminate variation in assay conditions. Reproducibility of the test kits was determined using human samples and controls in internal protocols. All analyses were performed at one point in time, therefore, coefficient of variance (CV) within run (n = 84 for sFer, n = 20 for sTRF and sTsfR) for the lower and upper range values were used.

Additional haematological indices were determined to obtain more comprehensive range of iron status indicators. Total iron binding capacity (TIBC) was calculated using the formula: TIBC (μmol/L) =25.1 × TRF (g/L) [26]. The upper threshold value of 1.5 was used for sTfR/log ferritin ratio (sTfR-F index) [27].

### Statistical analysis

Statistical tests were carried out using SPSS 16.0 (SPSS Inc., Chicago, Illinois, US). A significance level of P < 0.05 was used.

The normality of dependent variables was tested by Shapiro-Wilk test for baseline values for subjects’ age, BMI, training regime, iron status parameters, and exercise performance scores. The normal distribution, in terms of skewness and kurtosis, were assessed using 2 × standard deviation for both values. Log transformation was applied to variables that were not normally distributed. Boxplots were used to check for possible outliers.

Dietary confounders were assessed using baseline, mid-point and end means values applying repeated measures one-way ANOVA test.

Descriptive statistics and differences in mean values of investigated factors were tested by Pearson’s where no account for possible confounders is needed and Partial Correlation with the presence of confounders and Paired-sample t-test to determine the statistical significance effect of pre- and post-intervention. These tests were applied for the haematological data at baseline and end.

Baseline dietary intakes for macro- and micronutrients were compared to dietary recommendations using One-sample t-test.

## Results

### Participants’ characteristics

Characteristics of the participants are shown in Table 1. The mean age of participants was 32 years. The subjects were within the normal BMI range and engaged in regular exercise regime, mostly running.

**Table 1:** **Characteristics of study participants**

**ID**	**Age (yrs)**	**BMI (kg/m** ^**2**^ **)**	Main sports participating*	**Exercise regime (min/wk)**	**Running activity (min/wk)**	**VO**_**2max**_ **(ml/min/kg)**	**Compliance (bread slices/day)**
1	44	20	R (l-d)	380	300	46	6.2
2	29	23	R	170	110	40	3.0
3	20	22	R, V	540	360	38	3.6
4	36	21	R	120	60	36	1.4
5	28	22	R, SW	120	60	35	2.5
6	39	21	R (l-d)	480	480	42	5.0
7	31	25	R	160	100	35	5.5
8	26	24	D, R	180	30	42	3.5
9	38	27	R	200	150	34	2.1
10	33	28	R	160	60	33	5.0
11	33	25	R (l-d)	120	60	33	5.4
**Mean ± SD**	**32 ± 7**	**23 ± 2**		**239 ± 153**	**161 ± 150**	**38 ± 4**	**2403 ± 392**

Key: *R-running, R (l-d)- long-distance running, V- volleyball, SW-swimming, D-dancing.

Although the sample size was small (n = 11) the study employed stringent inclusion criteria and accounted for possible confounders, including dietary habits and training routine. The statistical tests for normality showed no significant anomalies for subjects’ age, BMI, nutrients intakes, exercise and blood iron parameters, suggesting the present study had overall normal variables distribution. No case of dietary underreporting was evident in the sample.

### Adequacy of diets before intervention

**Figure 1:**
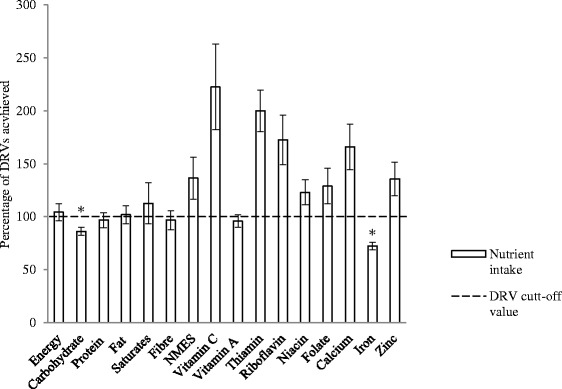
**Comparison of mean daily energy, macro- and micro-nutrient intake of female runners and dietary reference values.** Key: DRVs – Dietary Reference Values, TEI – total energy intake, NMES – non-milk extrinsic sugars. *represent significantly (*P* < 0.001) lower observed value compared to that of recommended intake. DRVs used: carbohydrate - 60% TEI ^(Ref)^, protein – 15% TEI ^(Ref)^, fat - <35% TEI ^(Ref)^, saturates - <10 TEI, fibre – 18g/day ^(Ref)^, NMES - <10% TEI, vitamin C – 40 mg/day, vitamin A - , thiamin - , riboflavin - , niacin - , folate, calcium – 700 mg/day, iron – 14.8 mg/day ^(Ref)^, zinc - .

**Figure 2:**
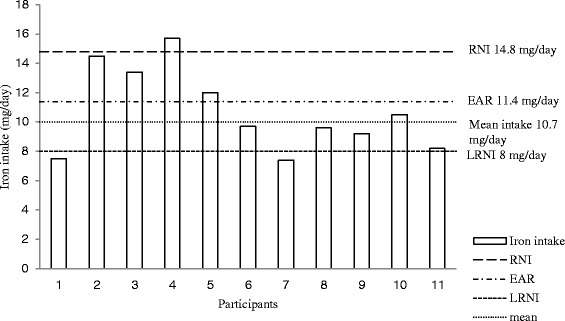
**Comparison of individual daily dietary iron intake in female runners and dietary reference values.** Key: LRNI - lower reference nutrient intake, EAR - estimated average requirements, RNI - reference nutrient intake.

Energy and nutrient intakes are presented in Figure 1. Female runners achieved the recommendations for total energy and protein. Fibre intakes were satisfactory. Total fat and saturated fatty acids intake was above the recommended value at the expense of carbohydrate intake, which was significantly lower than the recommendation. The intake of non-milk extrinsic sugars (NMES) was found to be above the recommended value of <10% total energy. Female runners exceeded the recommendations for Vitamin C, B-group vitamins, calcium and zinc. However, they did not meet recommendations for Vitamin A and showed significantly (*P* < 0.001) lower intake of iron compared to the recommendations (Figures 1 and 2).

### Changes in nutrient intake and iron status during intervention

The intake of total dietary iron increased significantly, from 10.7 to 18.5 mg/day (*P* < 0.05) as a result of the intervention. Teff bread provided 7.0 ± 3.3 mg iron a day which contributed to 45% of the daily recommendation value for dietary iron throughout the intervention period. Teff bread also contributed to 15% of carbohydrate, 16% of protein, 5% of fat and 31% of fibre daily dietary reference values as presented in Table 2.

**Table 2:** **Teff bread nutritional composition**

**Nutrients in Teff bread**	**Amount per 100 g**	**Total daily contribution to nutrients** ^**1**^	**Percentage daily contribution to DRVs** ^**1,2**^
	**Mean ± SD**	**Mean ± SD**	**Mean ± SD**
Carbohydrate (g)	43.2 ± 2.32	54.1 ± 16.14	15 ± 5.9
Protein (g)*	11.0 ± 0.02	13.8 ± 4.11	16 ± 5.8
Fat (g)*	3.7 ± 0.16	4.6 ± 1.38	5 ± 1.9
Fibre (g)*	4.5 ± 0.32	5.6 ± 1.68	31 ± 9.3
Iron (mg)*	5.62 ± 0.22	7.0 ± 3.32	45 ± 19.0

Key: DRVs – Dietary Reference Values, TEI – total energy intake.

*experimentally determined as previously described ^(Ref).^

^1^Based on reported consumption of average daily portion of 125 ± 37g of Teff bread by the participants.

^2^DRVs used: carbohydrate - 60% TEI ^(Ref)^, protein – 15% TEI ^(Ref)^, fat - <35% TEI ^(Ref)^, fibre – 18g/day ^(Ref)^, iron – 14.8 mg/day ^(Ref)^.

Although, haematological indices improved, there were no statistically significant differences observed in sTRF, sTsfR, sFer, TIBC and sTsfR-F index values before and after the intervention in the present study (Table 3).

**Table 3:** **Changes in haematological data pre- and post-intervention**

**Iron status parameter**	**Pre- intervention**	**Post-intervention**	**Change**	Intrinsic error (lower-upper range values)*
**Serum Transferrin (g/L)**	3.21 ± 0.6	2.97 ± 0.4	- 5.4%	0.86 – 0.77%
**Serum Transferrin Receptor (mg/L)**	3.43 ± 1.8	2.96 ± 1.3	- 12.8%	0.76 – 1.1%
**Serum Ferritin (μg/L)**	29.64 ± 20.9	33.40 ± 18.8	+ 5.4%	3.8 – 2.1%
**TIBC (μmol/L)**	81.2 ± 14.7	76.2 ± 9.8	- 6.2%	N/A
**sTfR-F index**	3.66 ± 3.7	1.94 ± 2.2	- 16.7%	N/A

Key: *Intrinsic error was determined as coefficient of variance (CV) within run (n = 84 for sFer, n = 20 for sTRF and sTsfR) for the lower and upper reference range values for all direct measurement haematological indices.

Table 4 shows correlations between changes in iron indices during intervention period, baseline iron status and other intervention variables. The changes in iron status parameters were correlated with reduced baseline iron tissue supply. Because of compliance issues, the intervention period varied across this cohort (4 to 6 weeks). The prolonged intervention period was positively correlated to improved iron tissue supply (ΔsTRF and ΔsTsfR). In terms of dietary iron changes as a result of the intervention, a significant (*P* < 0.05) positive relationship was observed between increased dietary iron and enlarged iron stores (ΔsFer).

**Table 4:** **Relationships between incremental haematological data and intervention variables**

**Changes in haematological indices**	**% change**	**Correlations with**	**r value,** ***P*** **value**
**ΔsTRF (g/L)**	-5.4	Baseline sTRF	r = - 0.7, *P* < 0.05
		Baseline sTsfR	r = - 0.8, *P* < 0.01
		Baseline TIBC	r = - 0.7, *P* < 0.05
		Baseline sTsfR-F index	r = - 0.8, *P* < 0.005
		No of days of intervention^1^	r = - 0.7, *P* < 0.05
**ΔsTsfR (mg/L)**	-12.8	Baseline sTsfR	r = - 0.8, *P* < 0.05
		Baseline sTRF	r = - 0.6, *P* < 0.05
		Baseline TIBC	r = - 0.6, *P* < 0.05
		Baseline sTsfR-F index	r = - 0.8, *P* < 0.005
		No of days of intervention*	r = - 0.6, *P* < 0.05
**ΔsFer (μg/L)**	+5.4	Δ sTsfR-F index	r = - 0.6, *P* < 0.05
		Δ dietary iron intake†	r = 0.8, *P* < 0.05

Key: sTRF - serum transferring, sTsfR - serum transferrin receptor, sFer - serum ferritin.

^*^partial correlations controlling for corresponding baseline haematological parameter.

†partial correlations controlling for changes in dietary calcium and vitamin C intake.

Δrepresents an incremental change.

*DRV cut-off value.

## Discussion

Despite the increased interest in nutrition amongst athletes and the well documented importance of a balanced diet in athletic performance, research suggests that many athletes might be consuming diets that are less than optimal [28,29]. This trend was also observed in the present study. The runners’ diets provided inadequate amounts of carbohydrate, whilst their intake of total fat, saturated fat and NMES were above the national recommendations (Figure 1). Most of micronutrient intakes were observed to be above reference nutrient intake (RNI), with the exception of Vitamin A and iron. The baseline iron intake of the present study’s cohort provided only 70% of RNI for iron [22]. This trend is in agreement with most of other researchers [30,31], who also reported most of macro- and micronutrient intakes to be above recommended values with the exception of iron. The mean intake iron in this study was 10.7 ± 2.7 mg/d. This corresponds to the other authors’ reported iron intakes of 11.0-12.2 mg/d for female athletes’ population [32,33] and in fact is similar to that of the general female population, which is reported to be at around 9.6 ± 3.0 mg/d [34]. In addition to this, the present study found that only 36% of runners reached estimated average requirement (EAR) of 11.4 mg/day, with 18% of them falling below the lower reference nutrient intake (LRNI) (Figure 2). This is similar to the levels (23%) reported for the general female population [34]. Taking into account that the iron requirement for female runners may be higher than general population [35], the baseline observations from the current study indicate that dietary iron intakes in this population are far from adequate.

As a result of the intervention, the dietary iron intake increased to 18.5 ± 3 mg/d by the end of the study. However, the improvements in iron status were observed at a statistically non-significant level (Table 3). Nevertheless, within-subject variation, measured as coefficient of variance, showed that iron tissue supply parameters (sTRF and sTsfR) improved at a notably greater increment when compared to the intrinsic error. This indicates that the changes were due to the intervention. However, this was not observed in iron tissue parameters (sFer) which indicates the change must have been notably influences by the within-subject variation.

The lack of significant change in iron status in the current study may be attributed to several factors. One plausible explanation would be that mean serum ferritin baseline value showed iron-repletion state (sFer 29.64 ± 20.9 μg/L). Researchers have demonstrated an inverse relationship between iron absorption and serum ferritin [36]. Hence, it may be possible that the subjects in this study had sufficient iron stores at baseline, which would explain the lack of significant effect of increased dietary iron intake on overall iron status. The majority of other iron therapy research studies recruited iron-depleted female runners (sFer < 20 μg/L) and used pharmacological dosage of iron supplementation, hence, it is difficult to compare them to the findings from current study. A study using a heme iron-based crisp bread dietary intervention in women of reproductive age showed a significant improvement in serum ferritin after 12 weeks of daily 35 mg of dietary iron intake [17]. The baseline serum ferritin levels of 24 μg/L were reported in Hoppe *et al.* study which suggest that participants may have had depleted iron stores. A longitudinal study by Tsalis *et al.* demonstrated no change in sFer levels of swimmers after 5 months of dietary intervention (26 mg/day) and a decrease in sFer after 6 months [13]. The participants in Tsalis *et al.* study showed similar iron storage values to the present study (sFer > 30 μg/L). Hence, similar baseline iron status of these studies cohorts may explain the lack of iron therapy effect in both studies.

Another reason for the lack of notable changes in iron status might be due to the dietary iron bioavailability. However, researchers suggest that around two thirds of dietary non-haem iron was incorporated into the red blood cells (RBC) after 2 weeks of consumption in participants with sufficient iron stores [37]. Therefore, non-haem iron derived from an iron-rich bread product in this study during 6-week intervention period would have been sufficient for the iron uptake and utilisation. Nevertheless, cereal products provide a less absorbable form of non-haem iron compared to animal sources, which contain haem iron [38]. Therefore, the lack of the effect on iron status may suggest lower iron bioavailability in Teff bread used in the present study.

Finally, the increase in dietary iron intake by 7 mg/day might not have been sufficient to see notable changes in iron status, especially as most of studies showing iron status improvements used therapeutic supplementation dosages (100 mg elemental Fe/day) [8,9]. Ishizaki *et al*. suggested that 4-week dietary intervention of 15 mg of iron a day increased the activity of δ-ALAD, an enzyme participating in red blood cells (RBC) turnover; however, this intervention did not increase any other haematological indices [14]. The dosage of dietary iron supplementation in the present study (mean of 7 mg/day from Teff bread, total intake of 18.5 mg Fe/day) is similar to the total dietary treatment of 15 mg/day by Ishizaki *et al*.

Despite the lack of significant changes in haematological indices, the study findings showed some important correlations between baseline iron status and favourable outcomes of the intervention. The iron status of iron-depleted participants showed the greatest improvements in haematological parameters during the intervention (Table 4). This was indicated by the correlations between changes in tissue iron supply parameters (sTFR and sTsfR) and the highest initial values of these haematological indices, showing inadequate iron supply at baseline. This can be explained by the homeostatic body iron metabolism control as the absorption of dietary iron is increased with compromised iron status [39,40]. This is in agreement with the findings of the present study in which female runners with a compromised iron status pre-intervention improved their haematological indices the most.

The increment in iron storage was positively correlated with changes in dietary iron intake, even when controlling for initial iron status (Table 4). This suggests that iron stores were increased more in runners, who increased their dietary iron intake the most regardless of their baseline iron status. This also indicates that dietary iron was incorporated into body iron stores. Hence, even a modest increase in dietary iron (from 10.7 to 18.5 mg/day) can provide beneficial effects on storage iron level.

## Conclusion

The present study revealed inadequate dietary iron intake in the cohort of female runners studied. In addition to this, over a third of the participants had serum ferritin levels that indicated an iron-deficiency state (serum ferritin <12μg/L). The dietary intervention showed significant improvements in total iron intake and a modest improvement in iron status.

Further research using larger groups of participants is needed to confirm if dietary intervention through a dietary change can significantly improve iron status and consequently exercise performance. Nevertheless, taking into account the research design and sample size limitations, the current study findings show a positive but modest improvement in iron status as a result of dietary iron intervention.

### Availability of supporting data

All the data from this research are available on your request.

## References

[1] Suedekum NA, Dimeff RJ (2005). Iron and the athlete. Curr Sports Med Rep.

[2] Zimmermann MB, Hurrell R (2007). Nutritional iron deficiency. Lancet.

[3] Beard J, Tobin B (2000). Iron status and exercise. Am J of Clin Nutr.

[4] Di Santolo M, Stel G, Banfi G, Gonano F, Cauci S (2008). Anemia and iron status in young fertile non-professional female athletes. Eur J Appl Physiol.

[5] Burke LM, Millet GE, Tarnopolsky MA (2007). Nutrition for distance events. J Sports Sci.

[6] Edgerton VR, Ohira Y, Hettiarachchi J, Senewiratne B, Gardner GW, Barnard RJ (1981). Elevation of hemoglobin and work tolerance in iron-deficient subjects. J Nutr Sci Vitaminol.

[7] Ohira Y, Edgerton VR, Gardner GW, Senewiratne B, Barnard RJ, Simpson DR (1979). Work capacity, heart-rate and blood lactate responses to iron treatment. Br J Haematol.

[8] Friedmann B, Weller E, Mairbaurl H, Bartsch P (2000). Effects of iron repletion on red blood cell volume and exercise performance. Med Sci Sports Exerc.

[9] Lamanca JJ, Haymes EM (1993). Effects of iron repletion on VO2_max_, endurance, and blood lactate in women. Med Sci Sports Exerc.

[10] Brownlie T, Utermohlen V, Hinton PS, Haas JD (2004). Tissue iron deficiency without anemia impairs adaptation in endurance capacity after aerobic training in previously untrained women. Am J Clin Nutr.

[11] Klingshirn LA, Pate RR, Bourque SP, Davis JM, Sargent RG (1992). Effect of iron supplementation on endurance capacity in iron-depleted female runners. Med Sci Sports Exerc.

[12] Peeling P, Blee T, Goodman C, Dawson B, Claydon G, Beilby J, Prins A (2007). Effect of iron injections on aerobic-exercise performance of iron-depleted female athletes. Int J Sport Nutr Exerc Metab.

[13] Tsalis G, Nikolaidis MG, Mougios V (2004). Effects of iron intake through food or supplement on iron status and performance of healthy adolescent swimmers during a training season. Int J Sports Med.

[14] Ishizaki S, Koshimizu T, Yanagisawa K, Akiyama Y, Mekada Y, Shiozawa N, Takahashi N, Yamakawa J, Kawano Y (2006). Effects of a fixed dietary intake on changes in red blood cell delta-aminolevulinate dehydratase activity and hemolysis. Int J Sport Nutr Exerc Metab.

[15] Lyle RM, Weaver CM, Sedlock DA, Rajaram S, Martin B, Melby CL (1992). Iron status in exercising women - the effect of oral iron therapy vs increased consumption of muscle foods. Am J Clin Nutr.

[16] Anschuetz S, Rodgers CD, Taylor AW (2010). Meal composition and iron status of experienced male and female distance runners. J Exerc Sci Fit.

[17] Hoppe M, Brün B, Larsson MP, Moraeus L, Hulthén LMoraeus L, Hulthén LMoraeus L, Hulthén LMoraeus L, Hulthén L (2013). Heme iron-based dietary intervention for improvement of iron status in young women. Nutrition.

[18] Alaunyte I, Stojceska V, Plunkett A, Ainsworth P, Derbyshire E (2012). Improving the quality of nutrient-rich Teff (*Eragrostis tef*) breads by combination of enzymes in straight dough and sourdough breadmaking. J Cereal Sci.

[19] Goldberg GR, Black AE, Jebb SA, Cole TJ, Murgatroyd PR, Coward WA, Prentice AM (1991). Critical-evaluation of energy-intake data using fundamental principles of energy physiology. 1.Derivation of Cut off limits to identify under-recording. Eur J Clin Nutr.

[20] Henry CJ (2005). Basal metabolic rate studies in humans: measurement and development of new equations. Public Health Nutr.

[21] Scientific Advisory Committee on Nutrition (SACN): *Dietary Reference Values for Energy*; 2011. https://www.gov.uk/government/uploads/system/uploads/attachment_data/file/339317/SACN_Dietary_Reference_Values_for_Energy.pdf (assessed January 2014).

[22] Department of Health Dietary Reference Values for Food, Energy and Nutrients for the United Kingdom (1991). Report of the Panel on Dietary Reference Values of the Committee on Medical Aspects of Food Policy.

[23] American Dietetic Association, Dietitians of Canada; American College of Sports Medicine (2009). American College of Sports Medicine position stand. Nutrition and athletic performance. Med Sci Sports Exerc.

[24] Schwellnus M (2008). The Olympic Textbook of Medicine in Sport.

[25] Tobin B, Beard J, Wolinsky I, Driskell JA (1997). Iron. Sports Nutrition.

[26] Vernet M (1993). Immunochemical assay of transferrin and iron saturation in serum. Clin Chem.

[27] Thomas C, Thomas L (2002). Biochemical markers and hematologic indices in the diagnosis of functional iron deficiency. Clin Chem.

[28] Hinton PS, Sanford TC, Davidson MM, Yakushko OF, Beck NC (2004). Nutrient intakes and dietary behaviors of male and female collegiate athletes. Int J Sport Nutr Exerc Metab.

[29] Papadopoulou SK, Papadopoulou SD, Gallos CK (2002). Macro- and micro-nutrient intake of adolescent Greek female volleyball players. Int J Sport Nutr Exerc Metab.

[30] Berning J, Troup JP, Van Handel P, Daniels J, Daniels N (1991). The nutritional habits of young adolescent swimmers. Int J Sport Nutr Exerc Metab.

[31] Hassapidou MN, Manstrantoni A (2001). Dietary intakes of elite female athletes in Greece. J Hum Nutr Diet.

[32] Pate RR, Miller BJ, Davis JM, Slentz CA, Klingshirn LA (1993). Iron status of female runners. Int J Sport Nutr Exerc Metab.

[33] Spodaryk K, Czekaj J, Sowa W (1996). Relationship among reduced level of stored iron and dietary iron in trained women. Physiol Res.

[34] Public Health England: *National Diet and Nutrition Survey. Results from Years 1, 2, 3 and 4 (combined) of the Rolling Programme (2008/2009 – 2011/2012)*; 2014. https://www.gov.uk/government/uploads/system/uploads/attachment_data/file/310995/NDNS_Y1_to_4_UK_report.pdf (assessed May 2014).

[35] Whiting SJ, Barabash WA (2006). Dietary reference intakes for the micronutrients: considerations for physical activity. Appl Physiol Nutr Metab.

[36] Hulten L, Gramatkovski E, Gleerup A, Hallberg L (1995). Iron-absorption from the whole diet - relation to meal composition, iron requirements and iron stores. Eur J Clin Nutr.

[37] Roughead ZK, Zito CA, Hunt JR (2002). Initial uptake and absorption of nonheme iron and absorption of heme iron in humans are unaffected by the addition of calcium as cheese to a meal with high iron bioavailability. Am J Clin Nutr.

[38] Hallberg L (1981). Bioavailability of dietary iron in man. Annu Rev Nutr.

[39] Beard J, Han O (2009). Systemic iron status. Biochim Biophys Acta.

[40] Frazer DM, Anderson GJ (2005). Iron imports. I. Intestinal iron absorption and its regulation. Am J Physiol Gastrointest Liver Physiol.

